# A transdisciplinary research agenda for resilience

**DOI:** 10.2471/BLT.26.295811

**Published:** 2026-07-27

**Authors:** Anne-Sophie Jung, Victoria Haldane, Cornelia Junghans-Minton, Matthew Harris, Paula de Castro Nunes, Paulo Victor Rodriges de Carvalho, Gustavo Matta, Alessandro Jatobá

**Affiliations:** a School of Politics and International Studies, University of Leeds, Leeds, England.; b Institute of Health Policy, Management and Evaluation, University of Toronto, Toronto, Ontario, Canada.; c Primary Care and Public Health, Imperial College London School of Public Health, London, England.; d Instituto de Saúde Coletiva, Universidade Federal Fluminense, Niterói, Brazil.; e Centro de Estudos Estratégicos Antônio Ivo de Carvalho, Fundação Oswaldo Cruz, Av. Brasil 4036, Office 1002 Manguinhos, 21040-361 Rio de Janeiro, Brazil.

Health system resilience is typically framed as the capacity to absorb, adapt and respond to acute shocks.^1^ The coronavirus disease 2019 (COVID-19) boosted global interest in health system resilience, and since 2020, an increasing body of work frames resilience as a goal of health system performance and a driver for public health and health system transformation. However, the concept of health systems resilience carries inherent tension. Even before the pandemic, debates emerged about its application to public health and health systems, with now ongoing discourse to better define and apply the concept of resilience, determine its scope and critically reflect upon what makes a health system resilient amid multiple and interacting crises.^2^ Today, critics warn that resilience risks functioning as a technocratic label that emphasizes adaptation while neglecting the underlying political determinants and inequitable distribution of resources, risks and capacities during periods of stress.^2^ Others maintain that resilience offers a distinct conceptual lens for understanding how systems navigate uncertainty and chronic stress, especially where instability is the norm.^2^

This article aims to set a conceptual agenda, drawing on an ongoing transdisciplinary collaboration between Fundação Oswaldo Cruz, University of Leeds, University of Toronto and Imperial College London. Our shared concern is that dominant framings of resilience, built around episodic preparedness, measurement frameworks and recovery from acute crises systematically miss the more persistent forms of resilience that constitute everyday life in health and social care systems.^3^ We argue that the conceptual centre of gravity in resilience research must shift from shock to the ordinary. We propose that the analytical work missing from current discussions is a clearer explanation of how chronic stressors interact with system functioning, not as isolated events to be repelled, but as accumulating pressure that demands continuous recalibration.

## Reframing resilience

Resilience understood as crisis response favours visibility, such as during pandemics, disasters or outbreaks. Less visible and more chronically consequential is what some have termed transilience, that is, long-term transformation under continuous stress.^2^ Stressors such as rising multimorbidity, antimicrobial resistance, ageing populations, climate-driven service strain, health workforce attrition and supply fragility are not episodic. These stressors are large-scale, gradually intensifying and arguably more consequential for population health than the acute disruptions for which preparedness frameworks were built.

To understand and conceptualize resilience one should study the mundane, ordinary or everyday uncertainties that health systems are confronted with.^3^ Doing so moves the notion of resilience away from its overt focus on acute shocks. Stressors like the rising number of patients with co-morbidities and chronic diseases or ageing populations have become common and insidious burdens to health care. The everyday, defined as the spatiality of situated, mundane and habitual practices,^4^ brings novel challenges to health care; challenges that are not acute, but are large-scale and long-term and which could have even more devastating effects for health outcomes than, for example, COVID-19.^5^

This everyday and transformative perspective on resilience directs attention beyond dramatic disruptions to focus on the relational, moral and infrastructural work required to sustain systems over time. This approach calls for examining how frontline health workers, that is, those who provide care directly to patients or communities rather than working in administrative, managerial or policy roles, negotiate uncertainty. The approach also calls for an understanding of system design and how it can support and receive feedback from the coping strategies of frontline health workers. Conceptualizing resilience around the ordinary shifts the analytical focus from how systems bounce back to the infrastructure and labour that sustain systems under stress. Resilience should not merely maintain the status quo but seek long-term, incremental transformation within systems constantly navigating structural inequities and political changes. Resilience entails the sustained capacity to influence and improve system outcomes to some degree.^3^ Without this ability, resilience becomes resignation.

If resilience is to mean something analytically beyond emergency response, research must trace chronic stressors which accumulate as drift, friction and demand creep, that is, pressures that recalibrate what counts as normal operation. Resilience-engineering scholarship offers useful vocabulary to do so. The gap between work-as-imagined (formal protocols, performance metrics, dashboards) and work-as-done (actual situated practice) is where everyday resilience lives.^6^ Frontline health workers continually translate inadequate resources into adequate-enough care; this translation is invisible to standardized metrics but essential for health system functioning. A meaningful resilience research agenda should examine how health systems continuously adapt to accumulating pressures over time, treating this interaction as a primary object of inquiry rather than as a precursor to crisis events.

## An example

Antimicrobial resistance exemplifies an ordinary stressor because it is not typically understood as a sudden crisis. Instead, antimicrobial resistance shapes prescribing practices, infection control, surveillance and procurement across every clinical encounter. Globally, antibacterial resistance was associated with an estimated 4.95 million deaths in 2019.^7^ Studying resilience through the antimicrobial lens requires examining how routine work is executed: how stewardship guidelines are interpreted under time pressure, how laboratory infrastructure and classification systems shape empirical prescribing and how informal mentoring by senior clinicians sustains good practice. These aspects are not auxiliary questions to antimicrobial control, but its foundation. The same logic extends to tuberculosis, mental health and the management of ageing populations. 

## Achieving comparable redesign

While equitable partnerships and co-production have become a focus of global health, we insist on the need for genuine learning from low- and middle-income countries, not as sites of data collection, but as sources of theoretical and practical insight.^8^ Health systems in many of these countries have long been characterized by chronic instability, informal workarounds and scarce resources. Yet these are precisely the conditions under which everyday resilience emerges.^7^^,^^9^

Health systems in low- and middle-income countries have long been places of everyday resilience under chronic constraint, and genuine learning must flow from these settings.^10^ The Community Health and Wellbeing Worker programme in the United Kingdom of Great Britain and Northern Ireland, modelled on Brazil’s *Estratégia Saúde da Família*, is one example of high-income country adoption of a design developed in a low- and middle-income country.^11^ Less commonly noted is that some high-income country systems are themselves redesigning around the ordinary. Singapore’s Healthier SG initiative, launched in 2023, re-anchors primary care around longitudinal general practitioner–patient relationships, personalized health plans and the HealthHub-linked National Electronic Health Records, explicitly to address chronic disease accumulation and ageing rather than acute episodes.^12^

## Political practice

Resilience is often discussed alongside health system strengthening, but the two should not be treated as synonymous. Health system strengthening typically operates as a normative improvement agenda, focused on building capacity, expanding coverage and enhancing performance against predefined goals. Resilience, by contrast, is not inherently an improvement framework. Resilience is an analytical and political lens that centres uncertainty, disruption and the distribution of adaptive capacities across actors and levels of the system.

We need to re-politicize resilience, resisting the technocratic tendency to frame it as a neutral and measurable attribute while downplaying the power relations and structural inequalities that condition system performance. Instead, we argue that resilience is not a neutral good. The contrary can be the case, when resilience becomes a political tool. What counts as resilience for one actor (continuity of service delivery) can come at the cost of burnout or moral injury for another (the frontline health worker absorbing the adaptation). The notion of resilience has for too long been built on ableist norms (that is, social expectations assuming that everyone can function in the same way) that risk perpetuating health inequities.^9^^,^^13^ Resilience inherits histories of austerity, colonial extraction and policy failure that pattern present vulnerabilities. In this light, resilience becomes not only an operational challenge but an ethical and political one, raising questions about who gets to define resilience, who bears its costs and whose knowledge counts in framing solutions.

In contexts of persistent uncertainty, the most consequential resilience question is no longer how systems bounce back but what holds them together in the day to day operations. The research agenda that follows is not about better dashboards and preparedness plans for the next pandemic. Rather, the agenda should focus on understanding how systems adapt under accumulating stress. The agenda should examine where and at what cost efforts to keep things going succeed or fail; it should draw lessons from systems that have long operated under chronic constraints and from those now redesigning themselves around persistent pressures. The next decade of resilience scholarship will be measured by how seriously it takes the ordinary.
